# Acute Microvascular Impairment Post-Reperfused STEMI Is Reversible and Has Additional Clinical Predictive Value: A CMR OxAMI Study

**DOI:** 10.1016/j.jcmg.2018.10.028

**Published:** 2019-09

**Authors:** Alessandra Borlotti, Michael Jerosch-Herold, Dan Liu, Dafne Viliani, Alessia Bracco, Mohammad Alkhalil, Giovanni Luigi De Maria, OxAMI Study Investigators, Keith M. Channon, Adrian P. Banning, Robin P. Choudhury, Stefan Neubauer, Rajesh K. Kharbanda, Erica Dall’Armellina

**Affiliations:** a Division of Cardiovascular Medicine, Radcliffe Department of Medicine, John Radcliffe Hospital, University of Oxford, Oxford, United Kingdom; b Department of Radiology, Brigham and Women’s Hospital and Harvard Medical School, Boston, Massachusetts; c Oxford Heart Centre, John Radcliffe Hospital, Oxford, United Kingdom

**Keywords:** acute myocardial infarction, magnetic resonance, myocardial blood flow, 6M, 6 month(s), CFR, coronary flow reserve, CMR, cardiac magnetic resonance, EF, ejection fraction, FPP, first-pass perfusion, IMH, intramyocardial hemorrhage, IMR, index of microvascular resistance, IS, infarct size, LGE, late gadolinium enhancement, LV, left ventricular, MBF, myocardial blood flow, MBF_cor_, myocardial blood flow corrected, MBF_CULPRIT_, average myocardial blood flow in the culprit territory, MI, myocardial infarction, MVO, microvascular obstruction, PPCI, primary percutaneous coronary intervention, STEMI, ST-segment elevation myocardial infarction, T_mn_, transit time at rest, T_2_W, T_2_-weighted, WT, wall thickening

## Abstract

**Objectives:**

This study sought to investigate the clinical utility and the predictive relevance of absolute rest myocardial blood flow (MBF) by cardiac magnetic resonance (CMR) in acute myocardial infarction.

**Background:**

Microvascular obstruction (MVO) remains one of the worst prognostic factors in patients with reperfused ST-segment elevation myocardial infarction (STEMI). Clinical trials have focused on cardioprotective strategies to maintain microvascular functionality, but there is a need for a noninvasive test to determine their efficacy.

**Methods:**

A total of 64 STEMI patients post–primary percutaneous coronary intervention underwent 3-T CMR scans acutely and at 6 months (6M). The protocol included cine function, T_2_-weighted edema imaging, pre-contrast T1 mapping, rest first-pass perfusion, and late gadolinium enhancement imaging. Segmental MBF, corrected for rate pressure product (MBF_cor_), was quantified in remote, edematous, and infarcted myocardium.

**Results:**

Acute MBF_cor_ was significantly reduced in infarcted myocardium compared with remote MBF (MBF_infarct_ 0.76 ± 0.20 ml/min/g vs. MBF_remote_ 1.02 ± 0.21 ml/min/g, p < 0.001), but it significantly increased at 6M (MBF_infarct_ 0.76 ± 0.20 ml/min/g acute vs. 0.85 ± 0.22 ml/min/g at 6M, p < 0.001). On a segmental basis, acute MBF_cor_ had incremental prognostic value for infarct size at 6M (odds of no LGE at 6M increased by 1.4:1 [p < 0.001] for each 0.1 ml/min/g increase of acute MBF_cor_) and functional recovery (odds of wall thickening >45% at 6M increased by 1.38:1 [p < 0.001] for each 0.1 ml/min/g increase of acute MBF_cor_). In subjects with coronary flow reserve >2 or index of myocardial resistance <40, acute MBF was associated with long-term functional recovery and was an independent predictor of infarct size reduction.

**Conclusions:**

Acute MBF by CMR could represent a novel quantitative imaging biomarker of microvascular reversibility, and it could be used to identify patients who may benefit from more intensive or novel therapies.

## Graphical abstract

**Figure:**
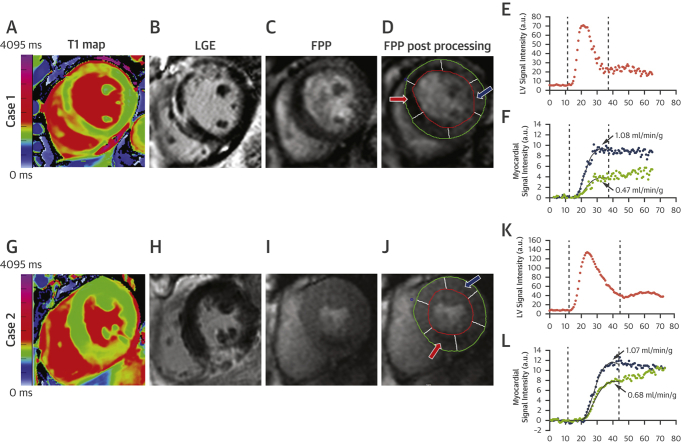

Despite the improvement in mortality rates following the introduction of primary percutaneous coronary intervention (PPCI), the incidence of heart failure post–myocardial infarction (MI) remains persistently high. Microvascular impairment despite successful restoration of epicardial coronary artery patency is associated with poor long-term recovery and outcome 1, 2. There is a recognized lack of innovative medical therapy targeting the microcirculatory function, partly because of an insufficient understanding of the underpinning pathophysiological mechanisms (3). Invasive coronary measurements such as the index of microvascular resistance (IMR) have emerging clinical utility for patients’ stratification at the time of PPCI, with abnormal values being more likely associated with microvascular obstruction (MVO) (4).

Although standard cardiac magnetic resonance (CMR) techniques such as late gadolinium enhancement (LGE) allow anatomic volumetric quantification of MVO and intramyocardial hemorrhage (IMH) as biomarkers of severe microvascular disease (5), they do not provide any insight into microvascular function. Currently there is no noninvasive imaging method to stratify patients at risk of developing MVO or to determine the dynamic changes in microvascular impairment at the time of PPCI.

First-pass perfusion (FPP) by CMR for assessment of myocardial blood flow (MBF) (6) has been extensively used to estimate abnormal perfusion in patients with chronic artery disease (7), but a systematic investigation of MBF in acute STEMI is lacking.

By using MBF CMR, we sought to investigate: 1) the degree of microvascular impairment and its longitudinal changes in relation to the severity of ischemic injury; and 2) the extent to which the acute microvascular dysfunction predicts 6-month (6M) infarct size (IS) and myocardial functional recovery. This may help establish CMR as a clinically useful tool to determine the crucial role played by the microvascular function in the MI healing process and potentially provide novel and specific imaging biomarkers to assess the clinical efficacy of cardioprotective strategies.

## Methods

### Patient study group

Survivors of acutely reperfused STEMI post-PPCI were prospectively recruited between October 2010 and March 2015. The study protocol was approved by the local ethics committee, and all patients gave written informed consent. Acute clinical management reflected contemporary practice and guidelines (Supplemental Appendix).

### Coronary physiology

Transit time at rest (T_mn_) and after hyperemia, coronary flow reserve (CFR), and IMR measurements were performed at the time of primary percutaneous coronary intervention (PPCI) in the infarct-related artery, as previously described (8) (Supplemental Appendix).

### CMR protocol

CMR was performed on a 3-T magnetic resonance scanner (either MAGNETOM TIMTrio or MAGNETOM Verio, Siemens Healthcare, Erlangen, Germany), acutely (1 to 3 days post-PPCI) and at 6M. The CMR protocol (details of the sequences can be found in the Supplemental Appendix) included functional cine imaging, tissue characterization techniques such edema T_2_-prepared steady-state free precession (SSFP) imaging (T_2_-weighted [T_2_W]), native shortened modified Look-Locker inversion recovery (ShMOLLI) T1 mapping, FPP at rest, and LGE. To track the first pass of a gadolinium-based contrast agent, 0.03 mmol/kg gadoterate meglumine (Dotarem, Guerbet, Villepinte, France) was injected at rest. LGE images were collected 10 to 15 min after the administration of an additional 0.1 mmol/kg of contrast agent. The inversion time was adjusted for nulling of remote normal myocardium.

Matching short-axis images covering the entire left ventricle were acquired using all techniques except for FPP, which was limited to 3 to 5 matching short-axis slices.

### CMR imaging analysis

CMR analysis is described in detail in the Supplemental Appendix.

**Figure 1:**
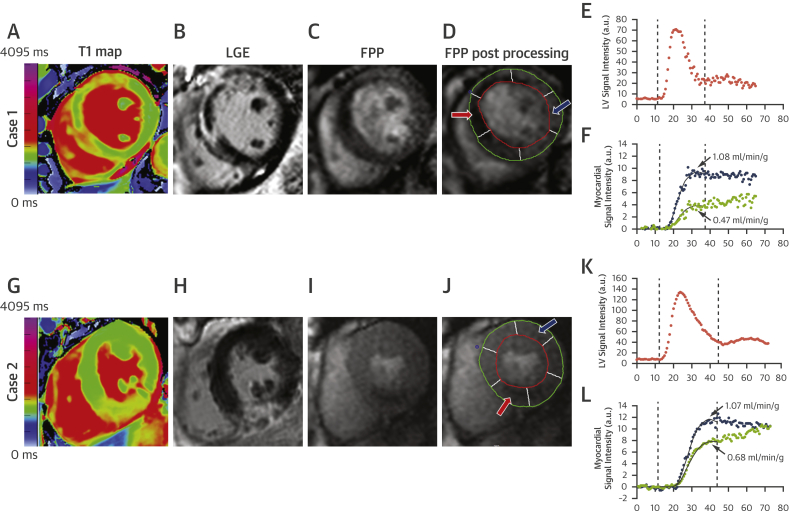
Perfusion Quantification for Assessment of Resting Segmental MBF **(A to F)** Case #1, anterior infarct with microvascular obstruction (MVO); **(G to L)** Case #2, inferior infarct with no microvascular obstruction. T1 maps and late gadolinium enhancement (LGE) images show the infarcted regions (**A and B,** anterior with microvascular obstruction; **G and H,** inferior) with hypoperfusion in the corresponding myocardium on first-pass perfusion (FPP) images **(C and I)**. From segmental quantification **(D and J)**, the myocardial tissue curves for myocardial blood flow (MBF) **(F and L)** demonstrated a reduced myocardial blood flow in the infarcted region **(red arrow, red curve)** compared with the remote myocardium **(blue arrow, blue curve). (E and K)** The arterial input function calculated in the blood pool. a.u. = arbitrary units; LV = left ventricular.

Both global and segmental analyses were performed on anonymized images using cvi42 software (Circle Cardiovascular Imaging Inc., Calgary, Canada) by 3 experienced operators (A.B., D.V., A.B.); all of the images were reviewed by an experienced CMR cardiologist (E.D.A). Apical slices affected by partial volume effects and slices where the outflow tract was visible were excluded from the study in all sequences. For segmental analyses, short-axis images were divided into 6 equiangular segments with the right ventricle–left ventricle junction as the reference point. The wall thickening (WT) analyses at follow-up were performed by an operator (A.B.) without knowledge of the baseline analyses. Functional recovery at 6M was assessed using a WT cutoff of 45% (9). Quantification of T_2_W edema and IS on LGE, both acutely and at 6M, was performed using a signal intensity threshold of 2 SD and 5 SD above the mean intensity of the remote reference region of interest (ROI), respectively, as previously described (10). When present, MVO and/or IHM were included in the measurements of left ventricular (LV) infarct or edema volume. Segmental tissue state was defined as remote if negative to LGE and on T_2_W, edematous if positive on T_2_W and negative to LGE, infarcted if positive to both (with a distinction between LGE 1% to 50% and LGE 51% to 100%), MVO (including segments with LGE >75% and MVO), and MVO in combination with IMH (including segments with LGE >75% with MVO and IMH) on the basis of acute LGE images. IS reduction was calculated as follows: (acute IS − 6M IS) / acute IS. Native T1 values were derived from short-axis T1 maps using in-house dedicated software MC-ROI (Interactive Data Language, version 6.1, Exelis Visual Information Solutions, Boulder, Colorado). Quantitative perfusion analysis to derive absolute MBF) (ml/min/g) was performed using an in-house MatLab software, as previously described 6, 7 (Figures 1A to 1L). MBF values were corrected for the heart rate–blood pressure product by dividing resting MBF by heart rate (in beats/min) × systolic blood pressure (mm Hg) / 10,000 (MBF corrected [MBF_cor_]). A per subject index of MBF (average MBF in the culprit territory [MBF_CULPRIT_]) was calculated averaging segmental MBF_cor_ of the culprit coronary artery territory.

### Statistical analysis

Statistical analyses were performed using IBM SPSS Statistics software (version 22.0, IBM Corporation, Armonk, New York) and the R statistical environment (version 3.4.1, R Project, Vienna, Austria). Normally distributed continuous variables are expressed as mean ± SD, and not normally distributed variables are expressed as median and interquartile ranges (first quartile to third quartile). The normality of the data was assessed visually using quantile-quantile plots and by the Shapiro-Wilk test. For normally distributed variables, comparison among groups were performed with unpaired Student’s *t*-tests, and comparisons within each group of follow-up versus baseline used paired Student’s *t*-tests. For variables such as %LGE (% of LV mass) that are not normally distributed, we used Wilcoxon rank sum and signed rank tests.

The lme4 package (11) for the R statistical environment was used to build linear models with mixed effects (LME) for the analysis of segmental MBF_cor_ acutely and over 6M follow-up (details in the Supplemental Appendix).

To investigate the predictive value of acute rest MBF_cor_ for LGE, WT at 6M, and WT<45% at 6M, we used Generalized Additive Models for Location Scale and Shape (GAMLSS) using acute rest MBF_cor_, LGE and MVO as predictors (details in the Supplemental Appendix). Spearman correlation coefficients were used to assess any relationships among segmental MBF_cor_, WT, and LGE and MBF_CULPRIT_ with invasive measurements and 6M myocardial functionality. Receiver-operating characteristic (ROC) analysis was performed to assess the diagnostic performance of MBF_CULPRIT_, LGE, and invasive measurements in the acute setting in predicting LV dysfunction (ejection fraction [EF] <50%) at 6M in patients with IMR <40 or CFR >2. Independent predictors of IS reduction from acute to 6M, in the same subgroup of patients, were assessed by adopting linear regression models. Variables with a p value <0.10 at univariable analysis were then entered into the multivariable models. The p values ≤0.05 were considered statistically significant.

## Results

Of 104 patients with STEMI who consented, 40 were excluded because of claustrophobia or technical issues (n = 12), bystander cardiomyopathy (n = 6), poor-quality image (n = 10), and declined follow-up scan (n = 12). A total of 64 patients underwent acute and follow-up scan. The measurement of IMR, CFR, and T_mn_ was feasible in 53 of 64 patients. Clinical and demographic baseline characteristics are shown in Table 1.

**Table 1:** Baseline Patient Characteristics (N = 64)

Age, yrs	60 ± 9
Sex, male:female	50:14
Risk factors	
Current smoker	18 (30)
Diabetes	4 (7)
Dyslipidemia	17 (28)
Hypertension	22 (36)
Family history of CHD	25 (41)
Peak troponin I (mg/ml)	65 (41–158)
Pain to balloon time (min)	179 (137–239)
Time from PPCI to CMR (days)	2 (1–3)
Culprit coronary artery	
LAD	29 (45)
RCA	29 (45)
LCx	6 (10)
No. of vessels diseased	
1	41 (67)
2	15 (25)
3	5 (8)
TIMI flow pre-PPCI	
0	50 (78)
1	6 (9)
2	7 (11)
3	1 (2)
TIMI flow post-PPCI	
0	0 (0)
1	0 (0)
2	4 (6)
3	60 (94)
Medications during PPCI	
GP IIb/IIIa inhibitor	8 (13)
Bivalirudin	42 (66)
Heparin	39 (61)
Clopidogrel	10 (16)
Medications post-PPCI	
Beta-blockers	61 (95)
ACE inhibitors	62 (97)
Statins	64 (100)
Aspirin	61 (95)
Diuretic	4 (8)
Nitrates	53 (83)
Clopidogrel	53 (83)
Ticagrelor	11 (22)
Invasive measurements post-PPCI (n = 53)	
IMR	32 (20–46)
CFR	1.7 (1.3–2.3)
T_mn_ (ms)	0.8 (0.4–1.2)

Values are mean ± SD, n (%), or median (first to third quartile).

ACE = angiotensin-converting enzyme; CFR = coronary flow reserve; CHD = coronary heart disease; CMR = cardiac magnetic resonance; GP = glycoprotein; IMR = index of microvascular resistance; LAD = left anterior descending coronary artery; LCx = left circumflex coronary artery; PPCI = primary percutaneous coronary intervention; RCA = right coronary artery; TIMI = Thrombolysis In Myocardial Infarction; T_mn_ = transit time at rest.

### CMR findings

The CMR findings are reported in Table 2. Acutely, all patients had positive edema, and only 1 had negative LGE; of 40 patients (63%) with MVO, 31 had also IMH. In 10 patients (i.e., 41 segments), myocardial edema persisted at 6M. Longitudinal native T1 changes relative to the degree of injury are also reported in Table 2 and Supplemental Figure 1A.

**Table 2:** CMR Findings

	Acute (n = 64)	6 Months (n = 64)	p Value
EF (%)	47 ± 9	54 ± 9	<0.001
EDV (ml)	153 ± 41	164 ± 42	0.001
ESV (ml)	83 ± 31	76 ± 31	0.002
LGE, 5 SD (LV%)∗	25 (14–32)	12 (8–19)	<0.001
Edema, 2 SD (LV%)∗	40 (33–48)	0 (0–0)	<0.001
MSI (%)∗	41 (31–60)	68 (58–78)	<0.001
MVO patients	40 (63)	—	—
MVO (g)∗	0.8 (0–2.6)	—	—
IMH patients	31 (48)	—	—
IMH (g)∗	0 (0–2.9)	—	—
MBF_CULPRIT_ (ml/min/g)	0.78 ± 0.14	0.87 ± 0.16	<0.001
Heart rate (beats/min)	67 ± 12	57 ± 8	<0.001
Systolic blood pressure (mm Hg)	113 ± 15	119 ± 18	0.002
Rate pressure product (beats/min·mm Hg/1e4)	0.75 ± 0.15	0.68 ± 0.15	<0.001
Segmental analysis			
WT (%)			
WT_remote_ (n = 276)	85 ± 34	88 ± 33	0.200
WT_edema_ (n = 228)	68 ± 29	80 ± 30	<0.001
WT_infarct_ (n = 406)	36 ± 27	48 ± 28	<0.001
WT_MVO_ (n = 36)	20 ± 17	32 ± 15	0.005
WT _MVO+IMH_ (n = 56)	15 ± 13	18 ± 14	0.062
T1 (ms)			
T1_remote_ (n = 150)	1,190 ± 55	1,183 ± 46	0.133
T1_edema_ (n = 152)	1,258 ± 64	1,197 ± 35	<0.001
T1_infarct_ (n = 306)	1,351 ± 86	1,239 ± 56	<0.001
T1_MVO_ (n = 31)	1,340 ± 70	1,281 ± 56	<0.001
T1_MVO+IMH_ (n = 47)	1,386 ± 82	1,280 ± 54	<0.001
MBF_cor_ (ml/min/g)			
MBF_remote_ (n = 276)	1.02 ± 0.21	1.08 ± 0.23	<0.001
MBF_edema_ (n = 228)	0.97 ± 0.18	1.03 ± 0.21	<0.001
MBF_infarct_ (n = 406)	0.76 ± 0.20	0.85 ± 0.22	<0.001
MBF_MVO_ (n = 36)	0.69 ± 0.16	0.76 ± 0.16	<0.001
MBF_MVO+IMH_ (n = 56)	0.59 ± 0.11	0.62 ± 0.10	0.080

Values are mean ± SD, median (first to third quartile), or n (%).

CMR = cardiac magnetic resonance; EDV = end-diastolic volume; EF = ejection fraction; ESV = end-systolic volume; IMH = intramyocardial hemorrhage; LGE = late gadolinium enhancement; LV = left ventricle; MBF_cor_, myocardial blood flow corrected; MBF_CULPRIT_ = average myocardial blood flow in the culprit territory; MSI = myocardial salvage index; MVO = microvascular obstruction; WT = wall thickening; 1e4 = 10^4^.

∗ Values were not normally distributed, and the p values then pertain to a paired Wilcoxon test.

### Longitudinal MBF_cor_ changes following acute MI

**Figure 2:**
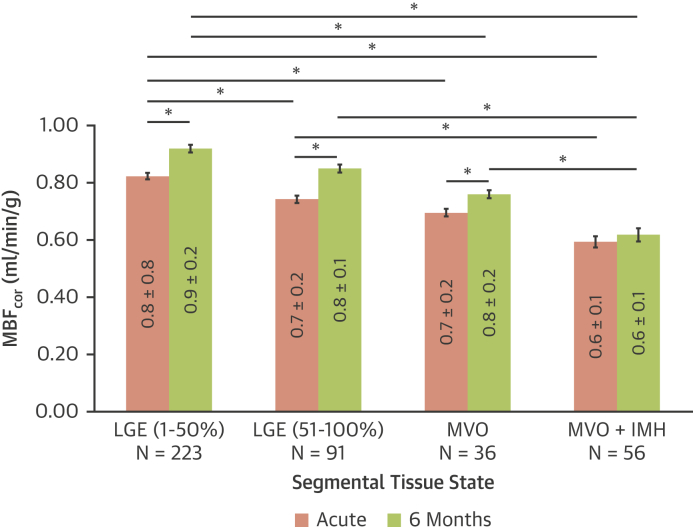
MBF_cor_ Changes Over 6M in Acutely Infarcted Myocardium Myocardial blood flow corrected (MBF_cor_) improves significantly at 6 months (6M) in all segments, with the exception of segments with an acute presentation with microvascular obstruction (MVO) and intramyocardial hemorrhage (IMH). **Bars** are SEM. Mean values in the **bars** are reported with SD. ∗p < 0.001. LGE = late gadolinium enhancement.

Acute MBF_cor_ decreased with worsening ischemic injury (p < 0.001) (Table 2, Supplemental Figure 1B), with the lowest MBF_cor_ values observed in infarcted segments with MVO and IMH. Over 6M, MBF_cor_ improved significantly in all segment classes, including the groups of segments with LGE and/or MVO. In segments with IMH, MBF_cor_ did not change significantly over time (p = 0.08) (Figure 2).

### Tissue state and severity of injury by T1 mapping as determinants of 6M MBF_cor_ changes

To determine the effect of tissue state on MBF_cor_ changes between the acute phase and 6M, we used a mixed-effects model for MBF_cor_, with tissue state and time point (acute, 6M), and their interaction as independent predictors. At the acute stage, edema, LGE, and LGE in combination with MVO were associated with a significant reduction of MBF_cor_ by 0.03 ml/min/g (p = 0.05), 0.18 ml/min/g (p < 0.001), and 0.38 ml/min/g (p < 0.001), respectively (Table 3). These model predictions are in close agreement with the differences in acute MBF_cor_ among tissue states that can be inferred from Table 2. At 6M, there was a significant, albeit small, overall improvement in MBF_cor_ (0.04 ml/min/g, p < 0.001), independent of tissue state. 6M LGE with edema had a by 0.11 ml/min/g worse effect than acutely (p = 0.002).

**Table 3:** Estimated Effect in Mixed-Effects Model for Mean Resting MBF_cor_, Acutely and at 6M

Multivariable Associations	Coefficient ± SE	p Value
Linear mixed-effect model for MBF with tissue state as predictors		
(Intercept)	1.00 ± 0.017	<0.001
Edema, acute	−0.03 ± 0.016	0.053
LGE	−0.18 ± 0.015	<0.001
LGE + MVO acute	−0.38 ± 0.020	<0.001
6M	0.04 ± 0.013	<0.001
Edema 6M	0.00 ± 0.075	0.989
LGE with edema 6M	−0.11 ± 0.037	0.002
LGE 6M	−0.19 ± 0.013	<0.001
Linear mixed-effect model for MBF_cor_ with T1 as predictor		
(Intercept)	0.90 ± 0.02	<0.001
T1 (100-ms change)	−0.09 ± 0.007	<0.001
6M	0.01 ± 0.01	0.409
T1: 6M	−0.05 ± 0.016	0.001

A 100-ms change of T1 rather than a 1-s change, was used here to estimate the effect size because it is of the same order of magnitude as the T1 differences at the acute stage between remote myocardium and regions with ischemic injury.

6M = 6 months; other abbreviations as in Table 2.

**Figure 3:**
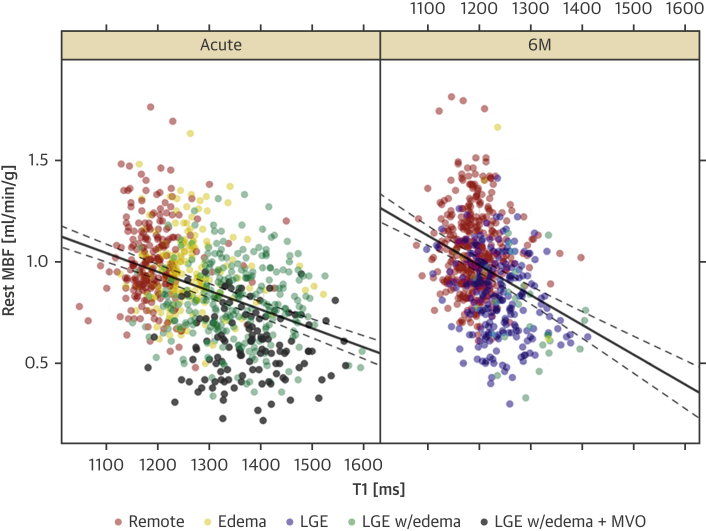
Relationship Between MBF_cor_ and Native T1 Over 6M The **lines** show the prediction from a linear mixed effects model for myocardial blood flow corrected (MBF_cor_), with native T1 and time point (with interaction term included) as predictors. The model included a random intercept by patient. The **dashed lines** show the 95th percentile ranges for the predictions from the linear mixed effects model. If regression coefficients for the effect of native T1 on myocardial blood flow are estimated for each tissue state, the association between myocardial blood flow and native T1 is significant only in the acute phase for late gadolinium enhancement with edema, and at 6 months (6M) for edema, late gadolinium enhancement (LGE), and late gadolinium enhancement with edema. MVO = microvascular obstruction.

Next, we investigated the association between MBF_cor_ and native T1 as quantitative marker of severity of injury. Acutely, a 100-ms increase in myocardial T1 was associated with a significant MBF_cor_ reduction of 0.10 ± 0.007 ml/min/g, whereas at 6M, an increase in native T1 by 100 ms was associated with a further MBF_cor_ reduction by 0.05 ± 0.02 ml/min/g (p = 0.001) (Table 3, Figure 3). We found a negative association between MBF_cor_ and T1 for all segments independent of the tissue state (Figure 3). Notably, if regression coefficients for the effect of native T1 on MBF are estimated for each tissue state, the association between MBF and native T1 is significant only in the acute phase for LGE with edema, and at 6M for edema, LGE, and LGE with edema.

### Relationship between MBF_cor_ and 6M infarct size

**Figure 4:**
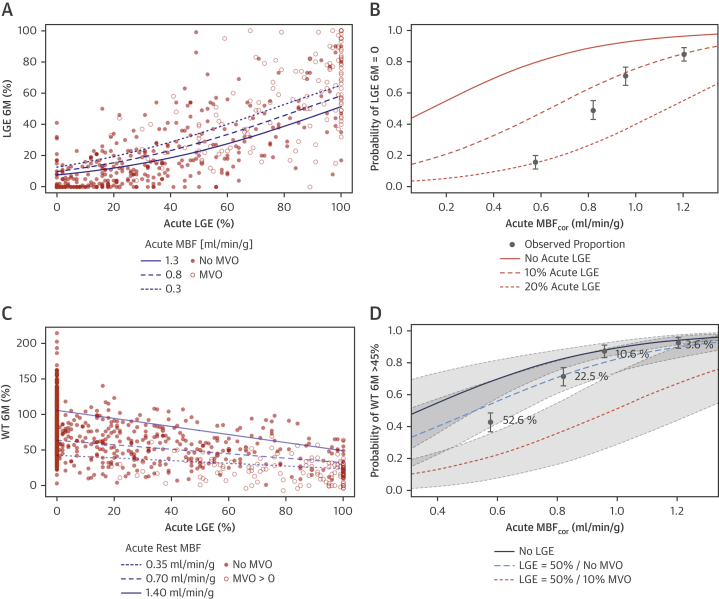
Predictive Value of MBF_cor_ for Final IS and 6M Functional Recovery **(A)** The **lines** show the predictions for acute myocardial blood flow corrected (MBF_cor_) equal to 0.3, 0.8, and 1.3 ml/min/g. **(B)** The observed proportions of segments with no 6-month (6M) late gadolinium enhancement (LGE) are shown by quartile of acute myocardial blood flow corrected, with 95% confidence intervals. The **lines** show the prediction for no 6-month late gadolinium enhancement as a function of acute myocardial blood flow corrected and acute late gadolinium enhancement of 0%, 10%, and 20%. **(C)** The continuous lines show prediction for acute myocardial blood flow corrected equal to 0.35, 0.70, and 1.40 ml/min/g. **(D)** The observed proportions of segments with wall thickening (WT) <45% are shown by quartile of acute myocardial blood flow corrected, with 95% confidence intervals. The **lines** show the predictions for wall thickening <45% as a function of acute myocardial blood flow corrected, and 0% and 50% late gadolinium enhancement, and with and without microvascular obstruction in the case of 50% acute late gadolinium enhancement. IS = infarct size.

Both acutely and at 6M, MBF_cor_ was strongly associated with LGE (Supplemental Figure 2A). Acute MBF_cor_ had a significant effect on 6M LGE, independent of acute LGE and MVO (p = 0.013) (Table 4, Figures 4A and 4B). As an example of point estimates from the model summarized in Table 4, we note that for segments with LGE of 50% and acute MBF_cor_ of 0.3 ml/min/g, the predicted IS at 6M (34%) was ∼6% larger than in segments with the same LGE and an acute MBF_cor_ of 0.8 ml/min/g (28% LGE at 6M) and ∼11% larger than in segments with same LGE and an acute MBF_cor_ of 1.3 ml/min/g (23% LGE at 6M) (Figure 4A). There was no evidence of an interaction between acute LGE and MBF_cor_ for predicting 6M LGE.

**Table 4:** Predictive Value of Acute MBF_cor_, LGE, and MVO for 6M LGE and WT (GAMLSS Model)

Multivariable Associations	Coefficient ± SE	t Value	p Value
GAMLSS for LGE 6M			
(Intercept)	−1.7 ± 0.209	−8.4	<0.001
Acute LGE (%)	0.025 ± 0.002	15.4	<0.001
Acute MBF_cor_ (ml/min/g)	−0.564 ± 0.228	−2.5	0.013
MVO (%)	0.017 ± 0.006	2.6	0.009
GAMLSS for WT 6M			
(Intercept)	74.147 ± 1.522	48.71	<0.001
Acute LGE (%)	−0.377 ± 0.042	−9.01	<0.001
Acute MBF_cor_ (ml/min/g) (centered)	60.066 ± 6.314	9.51	<0.001
MVO (%)	−0.944 ± 0.238	−3.97	<0.001
Acute LGE: acute MBF_cor_	−0.360 ± 0.151	−2.39	0.017

GAMLSS = Generalized Additive Models for Location Scale and Shape; other abbreviations as in Table 2 and 3.

In segments without LGE at 6M, 97% had no acute LGE, whereas the remaining 3% had a median acute LGE of 10.5%, which was resorbed over 6M. Conversely, 9% of segments without acute LGE showed a median LGE of 13% at 6M. The probability of no LGE at 6M varied significantly with acute MBF_cor_ and LGE, as shown in Figure 4B. The odds of no LGE at 6M increased by 1.4:1 for each 0.1 ml/min/g increase of acute MBF_cor_ (95% confidence interval: 1.21 to 1.58; p < 0.001), independent of acute LGE. Each 10% increase of acute LGE decreased the odds of no LGE at 6M by 1:4.7 (p < 0.001).

### Predictive value of acute MBF_cor_ for 6M segmental functional recovery

Acute MBF_cor_ and LGE are simultaneous, independent predictors of 6M WT (Table 4, Supplemental Figure 2B and 2C). Notably, our results show that in segments with intermediate LGE (Figure 4C), acute MBF_cor_ can account for the relatively large variability of 6M WT independent of the level of acute LGE; segments with intermediate LGE (i.e., 50%) and MBF_cor_ of 0.35 ml/min/g have a 15% reduction of WT 6M compared with segments with the same LGE and MBF_cor_ of 0.7 ml/min/g, and a 28% reduction compared with segments with same LGE and normal MBF_cor_ of 1.0 ml/min/g.

Acute MBF_cor_ impairment decreases the likelihood of 6M functional recovery (Figure 4D), both in a univariate model that includes only MBF_cor_ (p < 0.001) and in a multivariate model that also includes acute T1 (p = 0.001), acute LGE (p = 0.02), and MVO (p = 0.001) as additional predictors. In the multivariate model the odds of WT >45% at 6M increased by 1.38:1 (95% confidence interval: 1.29 to 1.48) for each 0.1 ml/min/g increase of acute MBF_cor_ (Table 5).

**Table 5:** Odds Ratios and 95% Confidence Intervals for Univariate and Multivariate Logistic Regression Model for WT at 6M >45% (GAMLSS), With Forest Plots for Univariate and Multivariate Regression Analysis

	OR	2.5% Percentile for OR	97.5% Percentile for OR	t Value	p Value
A. Univariate model					
0.1 ml/min/g MBF_cor_ change	1.793	1.62	1.98	11.46	<0.001
100 ms acute T1 change	0.282	0.22	0.36	−10.55	<0.001
10% LGE change	0.654	0.61	0.70	−13.08	<0.001
10% MVO change	0.026	0.01	0.06	−8.10	<0.001
B. Multivariate model					
Intercept	0.172	0.12	0.26	−8.52	<0.001
0.1 ml/min/g MBF_cor_ change	0.717	0.63	0.82	−4.86	<0.001
100 ms acute T1 change	1.667	1.22	2.29	3.17	0.002
10% LGE change	1.098	1.00	1.22	1.79	0.074
10% MVO change	4.437	1.92	10.27	3.48	<0.001


OR = odds ratio; other abbreviations as in Tables 2, 3, and 4.

### Additional predictive value of MBF in addition to invasive measurements for 6M EF and is reduction

Results for CFR, IMR, and T_mn_ post-PPCI are summarized in Table 1.

**Figure 5:**
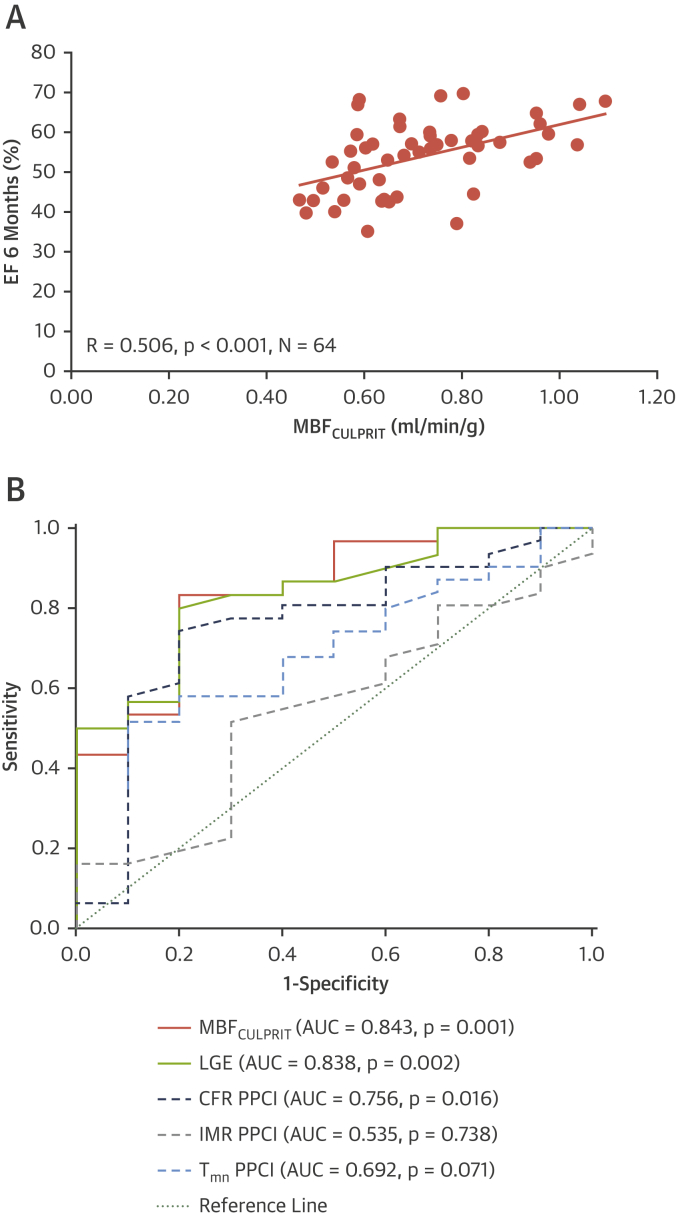
Relationship Between Acute MBF_CULPRIT_ and EF 6M **(A)** Per patient analysis. **(B)** Receiver-operating characteristic curves showing the diagnostic performance of acute average myocardial blood flow in the culprit territory (MBF_CULPRIT_) and late gadolinium enhancement (LGE), index of microvascular resistance (IMR), coronary flow reserve (CFR), and transit time at rest (T_mn_) in predicting left ventricular (LV) dysfunction (ejection fraction [EF] <50%) at 6 months (6M) in patients with index of microvascular resistance <40 or coronary flow reserve >2 at the time of primary percutaneous coronary intervention. AUC = area under the curve; PPCI = primary percutaneous coronary intervention.

MBF_CULPRIT_ correlated positively with 6M EF (R = 0.506, p < 0.001) (Figure 5A). When EF was simultaneously adjusted by acute LGE (p = 0.005) and acute EF (p < 0.001), the association remained significant (p = 0.02). In patients with IMR <40 or CFR >2 (N = 40), acute MBF_CULPRIT_ and LGE were equally strong predictors of EF <50% at 6M (area under the curve: 0.843, p = 0.001 for MBF_CULPRIT_; and area under the curve: 0.838, p = 0.002 for LGE) (Figure 5B). MBF_CULPRIT_ (p = 0.031) was independently associated with IS reduction (Table 6).

**Table 6:** Multivariable Associations Among Clinical Characteristics, Invasive Measurements at the Time of PPCI, and Infarct Size Reduction at 6M in Patients With IMR <40 or CFR >2

	IS Reduction From Acute to 6M			
	Univariable Analysis		Multivariable Analysis	
	Estimate Coefficient	p Value	Estimate Coefficient	p Value
Age (yrs)	−0.005	0.163	—	—
Male	0.016	0.861	—	—
Diabetes	−0.144	0.266	—	—
Hypertension	−**0.161**	**0.026**	−**0.175**	**0.012**
Hypercholesterolemia	−0.110	0.165	—	—
Active smoking	0.009	0.896	—	—
Family history of CHD	0.056	0.413	—	—
Pain to balloon time (min)	0.0001	0.152	—	—
LAD vs. non-LAD	0.108	0.116	—	—
TIMI pre	−0.038	0.131	—	—
TIMI post	0.114	0.474	—	—
CFR PPCI	**0.095**	**0.041**	0.201	0.195
IMR PPCI	−0.002	0.473	—	—
EDV (ml)	0.000	0.710	—	—
ESV (ml)	0.000	0.714	—	—
EF (%)	0.001	0.733	—	—
Acute IS (%)	−0.001	0.673	—	—
MBF_CULPRIT_ (ml/min/g)	**0.350**	**0.071**	**0.394**	**0.031**
MVO (yes/no)	−**0.141**	**0.037**	—	—
MVO (%)	−0.020	0.206	0.201	0.209
IMH (yes/no)	−0.105	0.124	—	—
IMH (%)	−0.008	0.388	—	—

IS = infarct size; other abbreviations as in Table 1, Table 2, Table 3.

## Discussion

The microcirculation plays a crucial role in infarct healing as the main supply conduit of oxygen and nutrients and as a delivery path for pharmacological treatments. An impairment in microvascular function is associated with a poor prognosis (12). To the best of our knowledge, this is the first CMR study using absolute MBF quantification to systematically estimate the longitudinal changes in microvascular function and predict functional recovery following STEMI.

The main findings of the study are as follows: 1) the acute microvascular dysfunction is strongly associated with the extent of the ischemic injury; 2) it is reversible, depending on the severity of the acute injury; 3) acute MBF_cor_, LGE, and MVO are simultaneous predictors of final IS; 4) MBF_cor_ has incremental predictive value for final IS and segmental recovery, compared with predictions that are based solely on acute LGE; and 5) in a lower-risk subgroup of patients (IMR <40 or CFR >2), acute rest MBF in the culprit territory may be superior to acute invasive measurements in predicting long-term recovery and may be an independent predictor of IS reduction. Taken together, these results suggest that quantifying myocardial blood at the acute stage leads to an improved prediction of segmental myocardial viability and wall motion at follow-up.

The lack of detailed understanding of the pathophysiological cascade leading up to irreversible microvascular damage in the context of ischemia-reperfusion injury has prevented adequate pharmacological treatment (3). Previous evidence by positron emission tomography or CMR stress testing demonstrated partial recovery of the vasomotor function over time 13, 14; however, such studies were performed in a subacute phase post-MI, and MBF was assessed in the myocardium pertaining to the culprit artery, thus leading to values likely not fully representative of the severity of injury. For the first time, our findings clearly show that the progressive deterioration of the acute microvascular function following ischemic injury is reversible even in severely damaged tissue. MBF normalization in areas with intermediate degree of ischemic insult is in line with pre-clinical evidence showing reversible endothelial damage and increased permeability of the microvessels at the time of reperfusion 15, 16. In contrast, for areas with MVO, the underlying mechanisms promoting functional recovery and infarct reduction are less clear. The infarcted myocardium, once thought to be “dead,” is actually a dynamic tissue undergoing an extensive process of remodeling, ultimately forming a core of scar, surrounded by neoangiogenesis in the infarct border zone 17, 18. No improvement in MBF was seen in infarcted areas with persistent edema at 6M. As shown recently, residual iron deposits at 6M following acute IMH resulted from severe acute microvascular impairment (19). Therefore, the correlation between persistent edema and microvascular dysfunction seems plausible and in line with putative mechanisms for edema in the presence of capillary leakage or hemorrhage.

In this study, we demonstrate for the first time the incremental role of microvascular function for final IS in segments with intermediate or lower LGE transmurality, independent of the extent of the acute LGE and MVO. The long-term prognostic value of even small amounts of LGE has been previously studied 20, 21. Our results show that the probability of having no 6M LGE is significantly affected by acute microvascular function, a finding suggesting that measures to restore microvascular function after MI may have longer-term benefits. Unexpectedly, we found that a small fraction of segments without LGE acutely were positive to LGE at 6M. There are at least 3 potential explanations for this finding: 1) a mismatch of short-axis positions imaged at different time points; 2) lower diagnostic performance of standard CMR techniques such as T_2_W and LGE compared with more accurate parametric maps to quantify ischemic injury; and 3) late development of necrotic tissue within an area at risk.

Finally, our study shows that in patients with preserved microvascular function as assessed by invasive measurements post-PPCI (i.e., IMR <40 or CFR >2), acute MBF estimates of microvascular function could provide a tool to stratify patients and predict LV remodeling. The predictive relevance of the changes in microvascular function early after PPCI are known (8): CMR MBF assessment could represent a valid alternative to invasive repeated measurements.

### Study limitations

The results of this study should be interpreted in light of some limitations. In this study, continuous variables such as MBF and native T1 represent averages for each myocardial segment. Any estimate of an effect modification of MVO in the relationship of native T1 and MBF is likely not representative of MVO itself because MVO in most segments represented a relatively small subarea of a myocardial segment. Our IMR measurements (median 32; interquartile range: 20 to 46) are comparable to those in previous studies 4, 22; however, we did not find a significant relationship between IMR and MVO/MBF_CULPRIT_. There may be several reasons for this, including the small sample size and the assessment of resting perfusion rather than stress perfusion 3 days after the invasive coronary measurements (8). Further larger studies are needed to corroborate our findings.

## Conclusions

Microvascular function estimated using CMR MBF has additional clinical and prognostic value beyond tissue characterization. In the context of novel potential therapeutic targets such as endothelial integrity, vascular permeability, or angiogenesis (23), MBF measurements could represent a useful noninvasive tool for risk stratification of patients with STEMI post-PPCI and to guide cardioprotective treatment.

Perspectives **COMPETENCY IN MEDICAL KNOWLEDGE:** Acute microvascular impairment is dynamic and reversible over time. Beyond EF and IS, acute microvascular function has incremental prognostic value for LV remodeling.

**TRANSLATIONAL OUTLOOK:** Further studies are required to determine whether adjunct therapy targeting microvascular function at the time of PPCI will improve patients’ outcomes.
