# Supplementary material for: Acute Microvascular Impairment Post-Reperfused STEMI Is Reversible and Has Additional Clinical Predictive Value: A CMR OxAMI Study
Source: JACC Cardiovasc Imaging. 2019 Sep;12(9):1783–93. doi: 10.1016/j.jcmg.2018.10.028 (PMC6718360; doi:10.1016/j.jcmg.2018.10.028)
Supplement: Online Data [file mmc1.docx]

**Supplemental Appendix**

**Patient population**

Patients were eligible if the onset of symptoms had been <12 hours before PCI and if they had ST-segment elevation of at least 0.1 mV in >2 contiguous limb leads or at least 0.2 mV >2 contiguous precordial leads. Patients with previous MI, previous revascularization procedure (coronary artery bypass grafts or PCI), severe heart valve disease, known cardiomyopathy, or hemodynamic instability lasting >12 hours after revascularization were not enrolled. Further exclusion criteria were contraindications to CMR, including implanted pacemakers, defibrillators, or other metallic implanted devices and claustrophobia. Acute clinical management was at the discretion of the responsible physician, with the intention to reflect contemporary practice and guidelines (including use of aspiration catheters; glycoprotein IIb IIIa receptor inhibitors, and high-dose clopidogrel loading).

Troponin I (TnI) was determined pre PPCI, and 6, 24, and 48 hours after onset of MI.

**Evaluation of coronary microcirculation**

Coronary physiology was invasively assessed placing a pressure wire in the distal third of the infarct-related artery.

The transit time (T_mn_) was calculated as a mean from three injections of 5 mL room temperature saline through the guiding catheter. T_mn_ was recorded at baseline and after induction of hyperaemia with intravenous adenosine infusion (140 μg kg^-1^ min^-1^ for 120 s) into the right femoral vein. Simultaneous measurement of mean aortic pressure (Pa, by guiding catheter) and mean distal coronary pressure (Pd, by pressure wire) was made in the resting and maximal hyperaemic states. CFR was calculated as the ratio of the transit times at baseline and hyperaemia (1, 2). IMR was calculated as the distal coronary pressure at maximal hyperaemia multiplied by the hyperaemic T_mn_ (1, 2).

**Cardiac Magnetic Resonance**

CMR was performed at 2 time points: within 3 days from PPCI and at 6 months (6M) on a 3 Tesla MR scanner (either MAGNETOM TIM Trio or MAGNETOM Verio, Siemens Healthcare, Erlangen, Germany). T2W was performed using a T2-prep-SSFP single shot sequence with surface coil correction (TE/TR = 1/ 4.1 msec; effective TE = 60 msec; flip angle = 90°; pixel size: 2.1 × 1.6 mm). SSFP cine images were acquired using retrospective gating (TE/TR = 1.4/3.2 msec; flip angle = 50°; pixel size: 1.6 × 1.6 mm). Two to three-fold accelerated parallel imaging (GRAPPA) was used to shorten the breath-hold. ShMOLLI T1 maps were generated from 5-7 SSFP images with variable inversion preparation time as described previously.(3) Typical acquisition parameters were: TE/TR=1.07/2.14 msec, flip angle=35°, FOV=340×255mm, matrix size=192× 144, 107 phase encoding steps, actual experimental voxel size = 1.8 × 1.8 × 8 mm, GRAPPA = 2, 24 reference lines, cardiac delay time TD =500 msec and 206 msec acquisition time for single image, phase partial Fourier 6/8.(3)

For rest FFP imaging, an ECG-gated T1-weighted fast gradient echo sequence was used (echo time, 1.04 ms; repetition time, 2 ms, saturation recovery time, 100 ms; voxel size, 2.1×2.6×8 mm3; flip angle, 17°) to acquire 3 to 5 short-axis images every heart beat. To track the first pass of a gadolinium-based contrast agent 0.03 mmol/kg; gadoterate meglumine, Dotarem, Guerbet, Villepinte, France) injected at rest. LGE images were collected 10-15 min after the administration of 0.1 mmol/kg contrast agent (gadoterate meglumine, Dotarem, Guerbet, Villepinte, France). The inversion time was adjusted for optimal nulling of remote normal myocardium.

**CMR imaging analysis**

*Global analysis*

Anonymised images were analysed using cvi42 software (Circle Cardiovascular Imaging Inc., Calgary, Canada) by experienced operators. LV volumes and EF were assessed from SSFP images. Quantification of oedema on T2W and infarct size (IS) on LGE was performed placing using a signal intensity threshold of 2SD and 5SD above the mean intensity of the remote reference region of interest (ROI) respectively as previously described (4), (5). When present, microvascular obstruction (MVO) and/or haemorrhage (IHM) were included in the measurements of LV infarct/oedema volume. The LV MVO percentage fraction was quantified by manual delineation of the hypointense areas within the LGE region (6, 7); IMH was defined as a hypointense area within the oedema having a mean signal intensity 2SD below the signal intensity of the periphery of oedema on T2W images.(8) Oedema and IS were also quantified at 6M. IS reduction was calculated as (acute LGE – 6M LGE)/acute LGE.

*Segmental analysis*

Short-axis images were divided in 6 equiangular segments with the RV-LV junction as reference point. Apical slices affected by partial volume effects and slices were the outflow tract was visible were excluded from the study in all sequences. Segmental IS, MVO, oedema and IMH were calculated as segmental percentage fraction. Depending on the presence or not of injury, segments were defined as remote if negative to LGE and T2W, oedematous if positive on T2W and negative to LGE, infarcted if positive to both. The percentage of wall thickening (WT) on a segmental basis was calculated as difference between systolic and diastolic thickness over diastolic thickness(9). WT was calculated in remote segments (WT_remote_), oedema only segments (WT_oedema_) and infarcted segments (WT_infarct_) as defined by T2W and LGE; functional recovery at 6M was assessed using a WT cut off of 45% (10). Segmental T1 values were derived from short-axis T1 maps using in-house dedicated software MC-ROI (Interactive Data Language, version 6.1, Exelis Visual Information Solutions, Boulder, Colorado). T1 was calculated in remote segments (T1_remote_), oedema only segments (T1_oedema_) and infarcted segments (T1_infarct_) as defined by T2W and LGE. Quantitative perfusion analysis was performed using an in-house MatLab software as previously described (11, 12). Absolute myocardial blood flow (MBF) in ml/min/g was calculated for each myocardial segment by Fermi deconvolution model of myocardial signal intensity curves with the arterial input using an in-house MatLab software as previously described (11, 12)^,^(13). MBF values were corrected for the heart rate –blood pressure product by dividing resting MBF by heart rate [in beats per minute] x systolic blood pressure [mm Hg]/10,000. (14) MBF_cor_ was calculated in remote segments (MBF_remote_), oedema only segments (MBF_oedema_) and infarcted segments (MBF_infarct_).

**Statistical analysis- GAMLSS**

Linear models with mixed effects (LME)

The LME model for MBF_cor_ included as fixed-effects the tissue-state (“remote”, “oedema” and “LGE”, with “remote” as base level for reference), the time-point (“acute”, “6M”, with “acute” as base level), their interaction, and MVO. A random intercept per patient was included in the model to account for the correlation of observations within subjects due to unobserved individual effects. Variance inflation factors (VIF) were calculated to assess collinearity of predictors. To explore the association between MBFcor and native T1, a linear mixed-effects model for MBFcor, with native T1 and time point (and term interaction) as predictors was implemented. The model included a random intercept by patient.

Generalized Additive Model for Location Scale and Shape (GAMLSS)

For the analysis of acute MBF as predictor of LGE at 6 months a Generalized Additive Model for Location Scale and Shape (GAMLSS) was used. The distribution of LGE at 6M, expressed as a fraction from 0 to 1 (corresponding to 0 to 100%), was represented in the GAMLSS by an inflated beta distribution, a mix of a continuous distribution on (0, 1) and the Bernoulli distribution, which gives non-negative probabilities to 0 and 1 (no LGE, and transmural LGE, respectively). The motivation for using a GAMLSS for this analysis is due to the markedly non-normal distribution of residuals if one assumes that LGE% is normally distributed. An examination of the histogram for LGE% also shows a relatively large probability for LGE=0, and for LGE > 0 the distribution of values is more akin to a beta distribution then a normal distribution. The probability of LGE = 0 at 6 months is modelled as linearly dependent on LGE and MBF_cor_ at the acute stage. The variance is modelled as varying linearly with acute LGE. The residuals from the GAMLSS model were normally distributed, in contrast to the residuals obtained with standard multi-variate linear regression, which assumes that LGE is normally distributed.

To investigate the predictive value of acute rest MBF_cor_ for WT at 6 month we used a (GAMLSS), and specified a normal distribution with lower-end truncation to account for the fact that systolic WT has a lower bound around zero.

WT at 6 months was modelled as linearly dependent on acute LGE, acute MBF_cor_, and MVO, and an interaction between acute MBF_cor_ and acute LGE was included in the model to account for a possible variation of the effect on acute MBF depending on the level of acute LGE. For prediction of dysfunctional segments at 6M (WT<45%) a GAMLSS logistic regression model was built using acute rest MBF_cor_, LGE and MVO as predictors.

**Results**


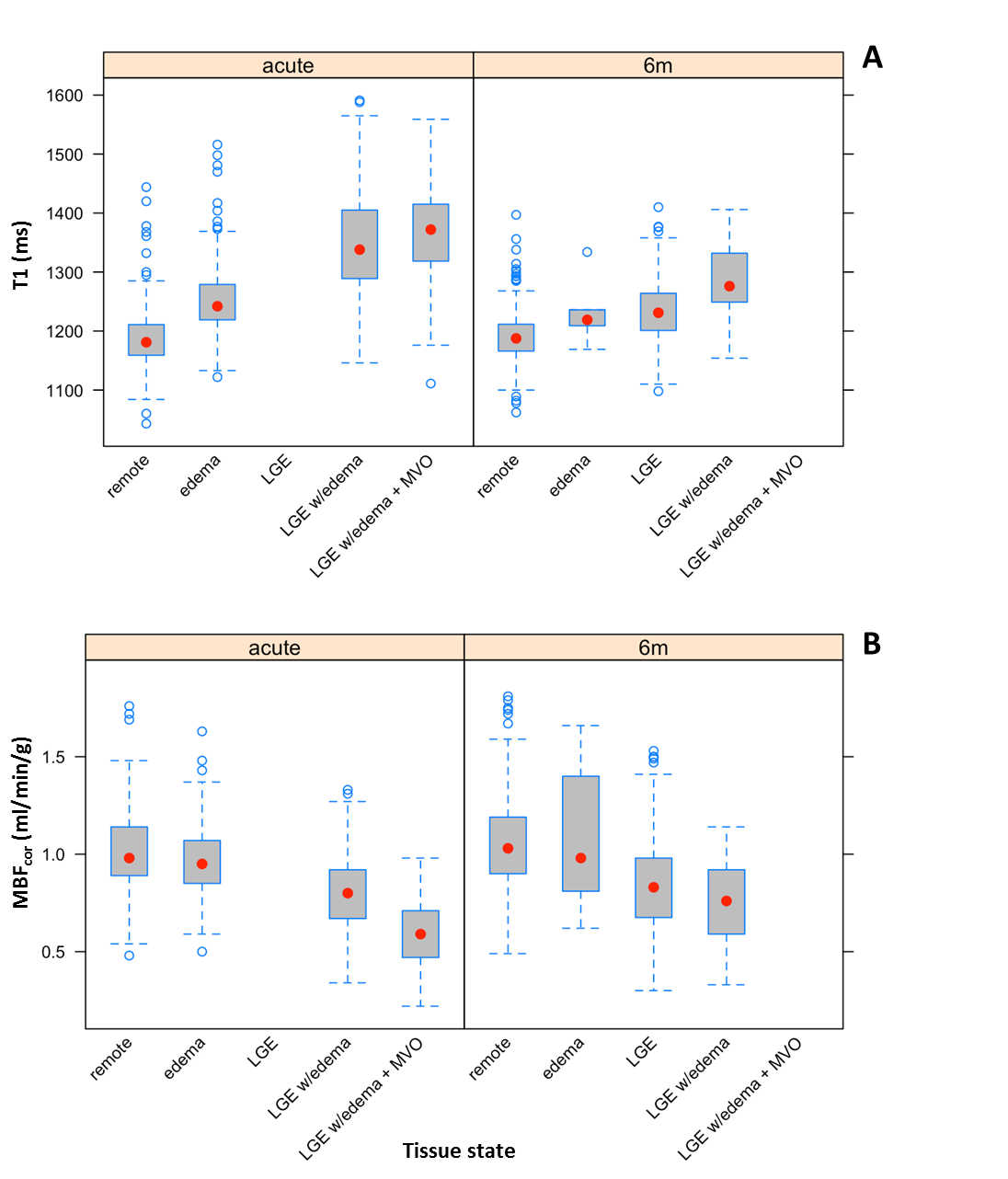


**Figure 1: Box-plots showing segmental native T1 (A) and segmental MBF_cor_ (B) according to the tissue state in acute and 6M**. Segments were classified as remote (T2W-/LGE-), oedema (T2W+/LGE-), LGE with oedema (T2W+/LGE+), LGE (T2W-/LGE+) and LGE with oedema and MVO. In the acute phase, none of the segments had LGE only and at 6M only 6 segments had oedema but not LGE. The red dot in the middle of the box is the median. The box represents the middle 50% of the data. The whiskers extend 1.5 x IQR from the box, where IQR is the inter-quartile range. Any data points beyond the whiskers are shown explicitly to highlight outliers.


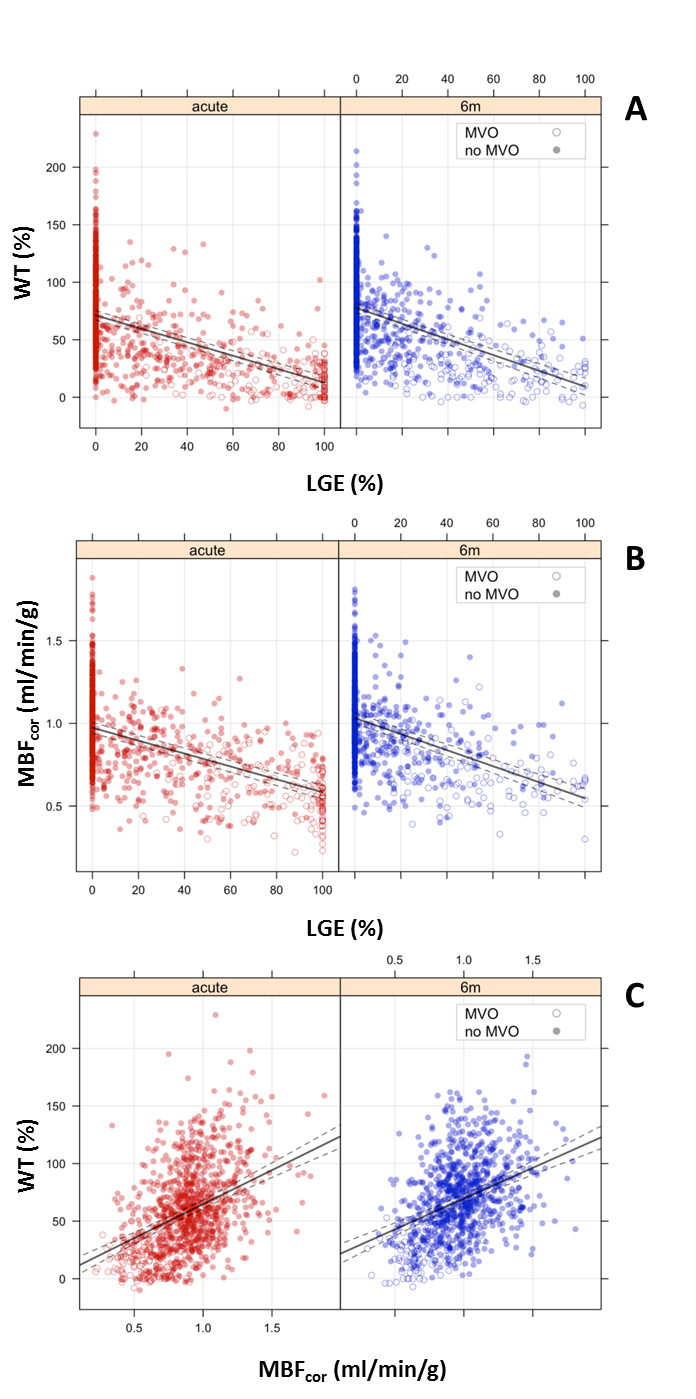


**A**


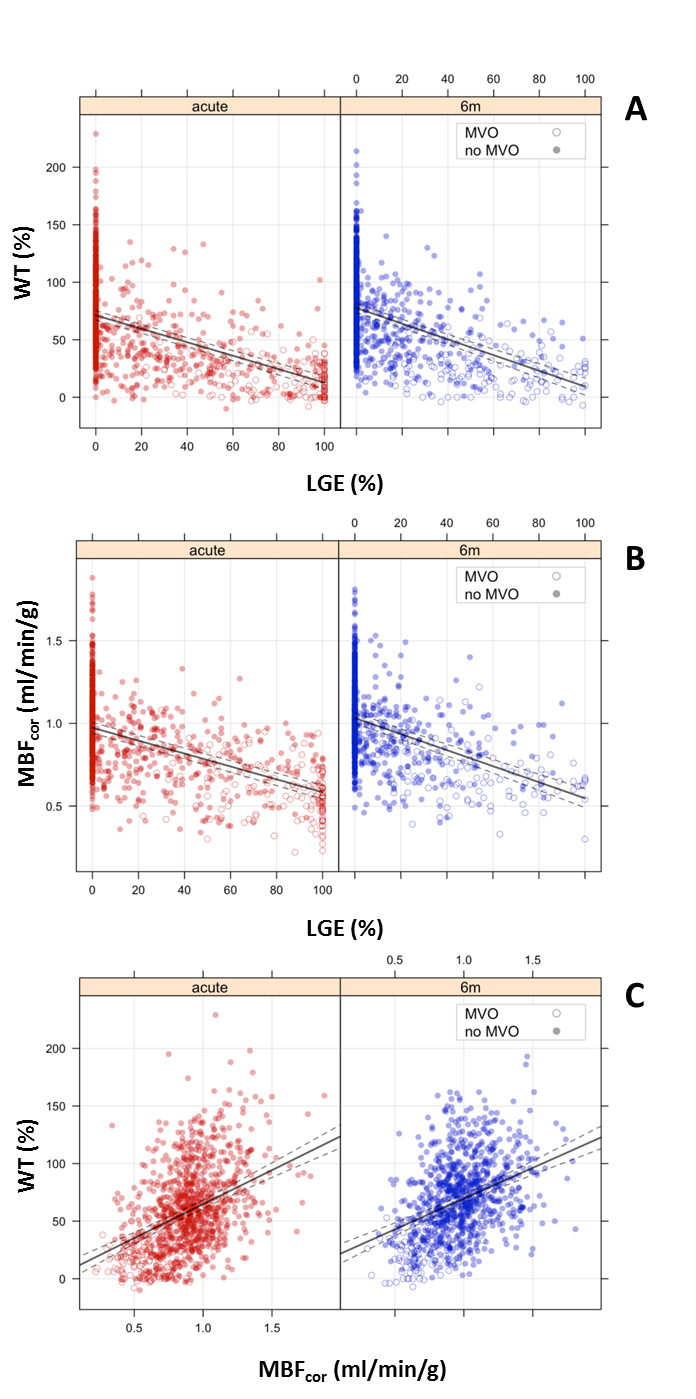


**B**


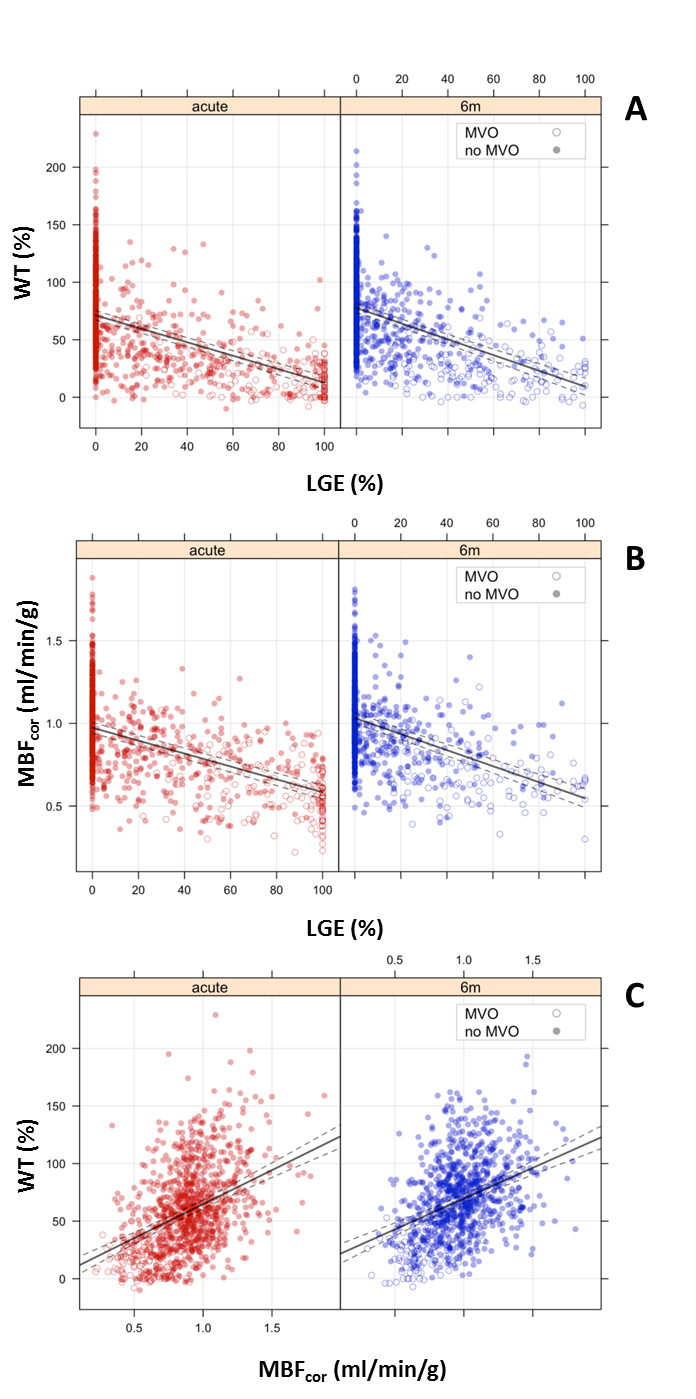


**C**

**Figure 2:** **Inter-Relation between Rest MBF_cor_ , WT and LGE at Acute and Chronic Stages.** The regression lines in the graphs are obtained without consideration of possible effects of MVO. A) effect of LGE on rest MBF_cor_ at baseline was ‑0.039±0.0018 ml/min/g (mean±SE; P<0.0001) per 10% change of LGE; rest MBF_cor_ was 0.06±0.0097 ml/min/g higher at 6 months compared to baseline (P<0.0001); and at 6 months, the effect of LGE on rest MBF_cor_ was larger compared to baseline (incremental effect of ‑0.01±0.003 ml/min/g per 10% change of LGE; P=0.002) B) effect of LGE on WT at baseline was -0.59±0.028 % per % change of LGE (P<0.0001); WT was 6.4±1.5% higher at 6 months compared to baseline (P<0.0001); and at 6 months, the effect of LGE on WT was larger compared to baseline (incremental effect of ‑0.098±0.045 % per % change of LGE; P=0.028) C) effect of rest MBF_cor_ on WT at baseline was 5.9±0.43 % per 0.1 ml/min/g change of MBF_cor_ (P<0.0001); WT trended 10.4±5.6 % higher at 6 months compared to baseline (P=0.06); and the effect of rest MBF_cor_ on WT was not significantly different at 6 months compared to baseline (P=0.335).

**References**

1. Cuculi F, Dall'Armellina E, Manlhiot C, et al. Early change in invasive measures of microvascular function can predict myocardial recovery following PCI for ST-elevation myocardial infarction. Eur Heart J. 2014;35:1971-80.

2. Ng MKC, Yeung AC, Fearon WF. Invasive Assessment of the Coronary Microcirculation: Superior Reproducibility and Less Hemodynamic Dependence of Index of Microcirculatory Resistance Compared With Coronary Flow Reserve. Circulation. 2006;113(17):2054-61.

3. Piechnik S, Ferreira V, Dall'Armellina E, et al. Shortened Modified Look-Locker Inversion recovery (ShMOLLI) for clinical myocardial T1-mapping at 1.5 and 3 T within a 9 heartbeat breathhold. J Cardiovasc Magn Reson. 2010;12(1):69.

4. Eitel I, Desch S, Fuernau G, et al. Prognostic Significance and Determinants of Myocardial Salvage Assessed by Cardiovascular Magnetic Resonance in Acute Reperfused Myocardial Infarction. J Am Coll Cardiol. 2010;55(22):2470-9.

5. Liu D, Borlotti A, Viliani D, et al. CMR Native T1 Mapping Allows Differentiation of Reversible Versus Irreversible Myocardial Damage in ST-Segment-Elevation Myocardial Infarction: An OxAMI Study (Oxford Acute Myocardial Infarction). Circ Cardiovasc Imaging. 2017;10(8):e005986.

6. Payne AR, Casey M, McClure J, et al. Bright Blood T2 Weighted MRI Has Higher Diagnostic Accuracy Than Dark Blood STIR MRI for Detection of Acute Myocardial Infarction and for Assessment of the Ischemic Area-at-Risk and Myocardial Salvage. Circ Cardiovascular Imaging. 2011,4:210-219.

7. Robbers LFHJ, Eerenberg ES, Teunissen PFA, et al. Magnetic resonance imaging-defined areas of microvascular obstruction after acute myocardial infarction represent microvascular destruction and haemorrhage. Eur Heart J. 2013;34: 2346–53.

8. Ganame J, Messalli G, Dymarkowski S, et al. Impact of myocardial haemorrhage on left ventricular function and remodelling in patients with reperfused acute myocardial infarction. Eur Heart J. 2009;30(12):1440-9.

9. Trieb T, Mayr A, Klug G, et al. Patterns of myocardial perfusion in the acute and chronic stage after myocardial infarction: A cardiac magnetic resonance study. European Journal of Radiology. 2012;81(4):767-72.

10. Baks T, van Geuns R-J, Biagini E, et al. Effects of Primary Angioplasty for Acute Myocardial Infarction on Early and Late Infarct Size and Left Ventricular Wall Characteristics. J Am Coll Cardiol. 2006;47(1):40-4.

11. Jerosch-Herold M, Swingen C, Seethamraju RT. Myocardial blood flow quantification with MRI by model-independent deconvolution. Medical Physics. 2002;29(5):886-97.

12. Jerosch-Herold M. Quantification of myocardial perfusion by cardiovascular magnetic resonance. J Cardiovasc Magn Reson. 2010;12(1):57-.

13. Jerosch-Herold M, Wilke N, Stillman AE, Wilson RF. Magnetic resonance quantification of the myocardial perfusion reserve with a Fermi function model for constrained deconvolution. Medical Physics. 1998;25(1):73-84.

14. Czernin J, Müller P, Chan S, et al. Influence of age and hemodynamics on myocardial blood flow and flow reserve. Circulation. 1993;88(1):62-9.
